# Diagnosis and treatment of the Ehlers-Danlos syndromes in China: synopsis of the first guidelines

**DOI:** 10.1186/s13023-024-03121-0

**Published:** 2024-05-13

**Authors:** Kexin Xu, Guozhuang Li, Zhihong Wu, Terry Jianguo Zhang, Nan Wu, On behalf of the Chinese Multi-Disciplinary Working Group on the Ehlers-Danlos Syndromes

**Affiliations:** 1 Department of Orthopaedic Surgery, Peking Union Medical College Hospital, Chinese Academy of Medical Sciences & Peking Union Medical College, No. 1 Shuaifuyuan, Beijing, 100730 China; 2 Beijing Key Laboratory for Genetic Research of Skeletal Deformity, Beijing, 100730 China; 3 Key Laboratory of Big Data for Spinal Deformities, Chinese Academy of Medical Sciences, Beijing, 100730 China; 4 Medical Research Center, Peking Union Medical College Hospital, Chinese Academy of Medical Sciences & Peking Union Medical College, Beijing, 100730 China; 5 State Key Laboratory of Complex Severe and Rare Diseases, Peking Union Medical College Hospital, Chinese Academy of Medical Sciences & Peking Union Medical College, Beijing, 100730 China

**Keywords:** Ehlers-Danlos syndromes, Multi-disciplinary team approach, Clinical guidelines, Diagnosis and treatment

## Abstract

**Background:**

The Ehlers-Danlos syndromes (EDS) are a group of rare hereditary connective tissue disorders. EDS is clinically and genetically heterogeneous and usually involves multiple systems. There are 14 subtypes of EDS with hallmark features including joint hypermobility, skin hyperextensibility, and tissue fragility. The clinical manifestations and their severity differ among the subtypes, encompassing recurrent joint dislocations, scoliosis, arterial aneurysm and dissection, and organ rupture. Challenges in diagnosis and management arise from the complexity of the disease, which is further complicated by its rarity. The development of clinical guidelines and implementation of coordinated multi-disciplinary team (MDT) approaches have emerged as global priorities.

**Main body:**

Chinese Multi-Disciplinary Working Group on the Ehlers-Danlos Syndromes was therefore established. Healthcare professionals were recruited from 25 top hospitals across China. The experts are specialized in 24 fields, including genetics, vascular surgery, dermatology, and orthopedics, as well as nursing care, rehabilitation, psychology, and nutrition. Based on GRADE methodology, the Guidelines were written by the Group supervised by methodologists, following a systemic review of all 4453 articles in PubMed published before August 9, 2023, using the search term “Ehlers Danlos”. A coordinated MDT approach for the diagnosis and management of EDS is highly recommended by the Group, along with 29 specific recommendations addressing key clinical questions. In addition to the treatment plan, the Guidelines also emphasize integrating support from nursing care, rehabilitation, psychology, and nutrition. This integration not only facilitates recovery in hospital settings, but most importantly, the transition from an illness-defined life to a more “normalized” life.

**Conclusion:**

The first guidelines on EDS will shorten the diagnostic odyssey and solve the unmet medical needs of the patients. This article is a synopsis of the full guidelines.

## Background

The Ehlers-Danlos syndromes (EDS) are a group of hereditary connective tissue disorders. EDS is characterized by joint hypermobility, skin hyperextensibility and tissue fragility, resulting in a spectrum of symptoms, ranging from joint dislocations, scoliosis, and poor wound healing, to pneumothorax, organ rupture, and arterial dissection. EDS is named after Edvard Ehlers and Henri­Alexandre Danlos, who independently reported the condition in 1901 and 1908 [1, 2]. Research in recent years has led to a growing appreciation of clinical and genetic heterogeneity in EDS. The classification of EDS has evolved from the 1988 Berlin Nosology’s 11 subtypes to the 1997 Villefranche Nosology’s 6 major subtypes, and expanded further in the 2017 International Classification, recognizing 13 subtypes, with 12 associated with 19 genes [3–5]. The 2017 Classification also published major clinical criteria, minor clinical criteria, and minimal criteria suggestive for diagnosis of these 12 subtypes and required molecular testing to confirm the diagnosis [5]. Clinical criteria were also revised for hypermobile EDS (hEDS), which is the only subtype with unknown genetic basis [5]. Classical-like type 2 was later introduced in the extended 2020 Classification, expanding the total number of EDS subtypes to 14 (Table 1) [6]. The cumulative prevalence of EDS was estimated to be 1 in 5,000 back in 2002 [7]. But the true prevalence of EDS is elusive, as the diagnostic criteria of hEDS has been significantly tightened while a number of patients remain undiagnosed or misdiagnosed.

**Table 1:** An overview of the 14 subtypes of EDS

Subtype	Abbreviation	Gene	Mode of Inheritance
Hypermobile	hEDS	Unknown	Unknown
Classical	cEDS	*COL5A1*, *COL5A2*, *COL1A1*	Autosomal Dominant
Vascular	vEDS	*COL3A1*, *COL1A1*	Autosomal Dominant
Arthrochalasia	aEDS	*COL1A1*, *COL1A2*	Autosomal Dominant
Brittle Cornea Syndrome	BCS	*ZNF469*, *PRDM5*	Autosomal Recessive
Cardiac Valvular	cvEDS	*COL1A2*	Autosomal Recessive
Classical-like	clEDS	*TNXB*	Autosomal Recessive
Classical-like Type 2	clEDS2	*AEBP1*	Autosomal Recessive
Dermatosparaxis	dEDS	*ADAMTS2*	Autosomal Recessive
Kyphoscoliotic	kEDS	*PLOD1*, *FKBP14*	Autosomal Recessive
Musculocontractural	mcEDS	*CHST14*, *DSE*	Autosomal Recessive
Myopathic	mEDS	*COL12A1*	Autosomal Dominant Autosomal Recessive
Spondylo-dysplastic	spEDS	*B3GALT6*, *B4GALT7*, *SLC39A13*	Autosomal Recessive
Periodontal	pEDS	*C1R*, *C1S*	Autosomal Dominant

### Challenges, Barriers, and Need for Clinical Guidelines

The challenges in diagnosis and management of EDS have long been recognized. Patients suspected or diagnosed with EDS have reported a significant, unmet medical need during both the diagnosis and management processes [8–10]. The average diagnostic delay from first symptom is around 14 years, which is likely to be consequential [11]. It not only delays appropriate treatment, but also places considerable psychological stress on the patients and families [12]. After receiving the diagnosis, patients see multiple doctors across specialties at different clinics, and report desires for multi-disciplinary care and support from patient community [8, 10, 13]. The uncoordinated care leads to worsened health outcomes and a significant decline in the quality of life, posing challenges to patients and families, as well as healthcare professionals.

The barriers are partially caused by the complex nature of EDS, limited awareness, and lack of practical guidelines. Despites the prominent features, the entire clinical presentation of EDS varies significantly across subtypes and among individuals. Some patients may present with recurrent multiple joint dislocations with widespread pain since childhood, which lead to diminished quality of life and disabilities. Others may remain unaware of the disorder until a sudden onset of life-threatening arterial dissection in their 20s. The manifestations of EDS also overlap with a number of disorders, such as Hypermobility spectrum disorders, Marfan syndrome, Loeys-dietz syndrome, Cutis laxa and Osteogenesis imperfecta [14, 15]. Therefore, the diagnosis and management of EDS typically requires coordinated multi-disciplinary team (MDT) approaches, which remain scant [16, 17]. In addition, limited knowledge and awareness among healthcare professionals further complicates the overall situation [16–18]. Efforts have been made to address the unmet clinical need, such as the free on-line program Ehlers-Danlos Society Extension for Community Health Care Outcomes (EDS ECHO), the outpatient EDS clinic at Mayo Clinic, USA, and the Ehlers-Danlos Syndrome Expert Panel and GoodHope EDS clinic at the Toronto General Hospital, Canada [16, 18, 19].

However, even at a multi-disciplinary clinic with experienced practitioners, the lack of clinical practice guidelines results in physician-dependent management plans that rely on theoretical and limited research evidence [20]. A recently published study conducted in the United States has reported an urgent need among medical providers for accessible consensus guidelines for care and management of patients with EDS [21]. However, creating high quality guidelines for rare disorders like EDS is challenging, largely due to the difficulties in gathering convincing evidence and gaining clinical experience [22].

## The development process

In order to shorten diagnostic odyssey and solve the unmet medical needs of the patients suspected of or diagnosed with EDS in China, Chinese Multi-Disciplinary Working Group on the Ehlers-Danlos Syndromes (the Group) was established, and Chinese Guidelines for Diagnosis and Treatment of the Ehlers-Danlos Syndromes (the Guidelines) were published in December 2023 [23].

Members of the Group are healthcare professionals recruited from 25 leading hospitals across China. They are specialized in 24 diverse fields such as genetics, vascular surgery, dermatology, orthopedics, nursing care, rehabilitation, psychology, and nutrition. The Group is committed to providing care for patients and families affected by EDS through an MDT approach and bring education and awareness into and beyond the medical community. Additionally, the Group aims to integrate basic, clinical, and translational research, paving the way to precision medicine for EDS. Following the publication of the Guidelines, the Group will develop specific versions of guidelines for primary care physicians, emergency medicine physicians, as well as patients and families.

The Guidelines were created based on the GRADE methodology and supervised by methodologists, following a systematical review of 4453 articles in PubMed published before August 9, 2023, using the search term “Ehlers Danlos.” The Guidelines are open access and publicly available. The Guidelines has been registered on the Practice guideline REgistration for transparency on November 02, 2023, with Registration Number PREPARE-2023CN840.

## The recommendations

**Fig. 1:**
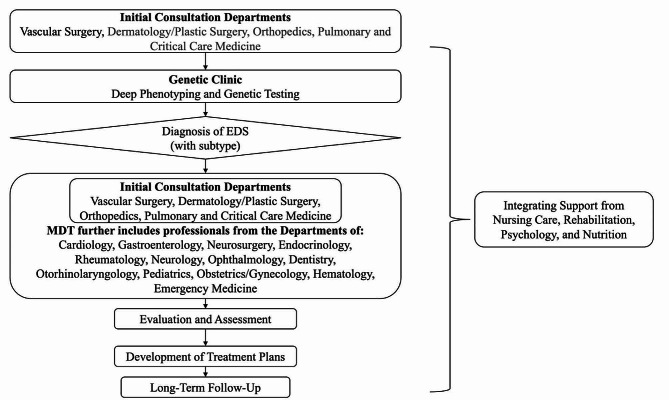
The multidisciplinary approach to the diagnosis and management of the Ehlers⁃Danlos syndromes (Translated from the Guidelines) [23]

The Guidelines highly recommend a coordinated MDT approach in both diagnosing and managing EDS, alongside 29 specific recommendations addressing crucial clinical questions encountered across 23 departments. The Guidelines recognize the Departments of Vascular Surgery, Dermatology/Plastic Surgery, Orthopedics, and Pulmonary and Critical Care Medicine as the “Initial Consultation Departments” as patients with EDS are more likely to seek initial consultation from physicians in these departments. Identifying features suggestive of EDS during the initial visit facilitates a prompt and accurate diagnosis. The Guidelines also emphasize the collective integration of nursing care, rehabilitation, psychology, and nutrition. The Guidelines aim not only to aid the recovery in hospital settings but more significantly, to the transition from a life defined by illness to one that is more “normalized.” The flowchart of the MTD approach has been summarized in Fig. 1 [23].

### MDT approach during diagnosis

The current focus of MDT approaches primarily centers on the management stage after diagnosis. But equal attention should be given to an MDT collaboration between geneticists and physicians from different departments during the diagnostic phase. Performing a comprehensive evaluation at individual level rather than organ level offers a broader understanding of the condition and markedly expedites timely diagnosis.

An MDT approach is recommended for the medical assessment of patients clinically suspected to have EDS. The clinical diagnostic criteria for each subtype of EDS were published in the 2017 International Classification and the extended 2020 version [5, 6]. It largely relies on physician’s personal experience and medical judgement since there is no test with high sensitivity or specificity. Patients suspected with EDS, however, usually present with a myriad of symptoms. Many of the symptoms, such like joint hypermobility, scoliosis, hypotonia, mitral valve prolapses and retinal detachment, may not be EDS-specific. Additionally, symptoms like wide-spread chronic pain may have both physical and psychological bases. The considerable overlapping features between subtypes and with other disorders further exacerbate the diagnostic intricacies. The Guidelines recommend physicians, especially dermatologists, pediatrician, or other physicians working in “Initial Consultation Departments”, to be aware of features that may not related to primary complaints but are indicative of a diagnosis of EDS. The features include joint hypermobility, skin hyperextensibility and fragility, characteristic facial features, and organ or vessel fragility. And then, an MDT approach is recommended for further assessment, differentiation of similar conditions, and identification of patients for genetic testing. As for hEDS, the only subtype without known genetic basis, the diagnosis relies solely on clinical evaluation. An MDT approach is particularly crucial for this group of patients to prevent misdiagnosis, as well as overdiagnosis, which may lead to unnecessary medical interventions and psychological burden.

The Guidelines also emphasize an MDT collaboration during the genetic testing stage to improve outcomes. The confirmation of the diagnosis in most of the subtypes requires genetic testing, but discrepancies between the clinical and molecular diagnosis have been observed [24, 25]. Besides, receiving a negative or inconclusive genetic test result is not uncommon. Deep phenotyping has been demonstrated an important role in improving diagnostic yield [26–30]. Physicians specialized in relevant field are recommended to participate in the pre-testing phenotyping, collaborating and communicating under an MDT framework, to provide accurate phenotypic description. During data analysis, geneticists may need support from clinicians to further evaluate and classify the pathogenicity of specific variants through methods such as reverse phenotyping. This MDT approach is critical in reducing the rate of false-negative results and the numbers of the variants of unknown significance.

After arriving at a diagnosis, the announcement is usually difficult for both the patients and their families. Psychologists hold a key position helping the patients and families in coping with their emotions, coming to terms with the diagnosis and transition into the management stage.

### The collective integration of nursing care, rehabilitation, psychology, and nutrition

The collective integration of nursing care, rehabilitation, psychology, and nutrition within an MDT approach is increasingly acknowledged as crucial for optimizing patient care, especially in complex disorders requiring holistic and multidisciplinary interventions. Undoubtedly, the collective expertise of professionals from these departments develops specialized and individualized plans, significantly expediting the recovery process within the hospital setting. Moreover, the Guidelines emphasize its key role in assisting patients and families effectively manage EDS on a day-to-day basis at home and live life to the fullest.

Some patients with EDS may often encounter joint instability, hypotonia, or postural tachycardia syndrome (PoTS), placing them at an increased risk of falls. It is recommended to implement home adaptations and various preventive strategies, including gradual postural changes, to minimize the risk of falls at home. Frequently, family caregivers feel unprepared and lack knowledge. Nurses can provide education about the disorder, assist in developing caregiving skills, and design individualized home care plan.

Rehabilitation for patients with EDS typically involves long-term participation. Rehabilitation professionals develop personalized clinic- or home- based rehabilitation plans to address various challenges such as joint instabilities, hypotonia, joint pain, reduced bone mineral density, shortness of breath, writing difficulties, and PoTS-related symptoms. Patient-centered self-management rehabilitation interventions aim to restore and improve daily functioning, eventually promoting independence in daily living activities.

Psychological interventions help manage chronic pain, a frequently reported challenge impacting daily lives. Moreover, chronic conditions like EDS may cause tremendous life changes, interfere with physical and social function, and shape the feelings. But it’s crucial to understand that the life is not solely defined by the diseases. Psychosocial adjustments address the fear and anxiety associated with uncertainties, such as the potential decline in abilities and sudden death. Additionally, EDS impacts the wellbeing of the families. Coping strategies not only help alleviate stress and tension for caregivers, but also provide support to healthy siblings who may feel overlooked.

Underweight and overweight are both frequently observed in patients with EDS. The complexity necessitates individualized nutritional care plans created by nutrition experts. From dietary supplements and modifications to eternal and total parenteral nutrition, home nutritional support serves as the foundation for treating symptoms and maintaining daily function.

One of the limitations of the Guidelines is that because EDS is a rare disease and there are few studies on relevant populations, it is difficult to form high-quality evidence for the research questions raised in the Guidelines. Therefore, the recommendations in the Guidelines are mainly recommendations given by multidisciplinary experts based on limited research evidence and their own clinical experience.

## Conclusion

We believe that the establishment of the Group and the publication of the Guidelines will substantially enhance the quality of life for patients and families affected by EDS. The Guidelines not only offer valuable resources for clinicians but also marks a significant step forward in deepening the understanding of EDS within and beyond the medical community.

In the future, the Group will continue gathering compelling evidence for updated versions of the Guidelines. Looking ahead, the Group will further strengthen the collaboration between practitioners and researchers, fostering an evolution towards precision medicine in EDS.

## Data Availability

The original Chinese version of the Guidelines is available at https://jrd.chard.org.cn/cn/article/doi/10.12376/j.issn.2097-0501.2023.04.013.

## Abbreviations

EDS: Ehlers-Danlos syndromes
MDT: Multi-Disciplinary team
hEDS: hypermobile Ehlers-Danlos syndrome
EDS ECHO: Ehlers-Danlos Society Extension for Community Health Care Outcomes
PoTS: Postural tachycardia syndrome
